# Bringing analysis of gender and social–ecological resilience together in small-scale fisheries research: Challenges and opportunities

**DOI:** 10.1007/s13280-016-0814-5

**Published:** 2016-09-10

**Authors:** Nozomi Kawarazuka, Catherine Locke, Cynthia McDougall, Paula Kantor, Miranda Morgan

**Affiliations:** 1 International Potato Center (CIP), International Center for Tropical Agriculture (CIAT), Hanoi, Vietnam; 2 School of International Development, University of East Anglia, Norwich, NR4 7TJ UK; 3 WorldFish, 10670 Penang, Malaysia; 4 Penang, Malaysia

**Keywords:** Gender, Interdisciplinarity, Small-scale fisheries, Social–ecological resilience

## Abstract

The demand for gender analysis is now increasingly orthodox in natural resource programming, including that for small-scale fisheries. Whilst the analysis of social–ecological resilience has made valuable contributions to integrating social dimensions into research and policy-making on natural resource management, it has so far demonstrated limited success in effectively integrating considerations of gender equity. This paper reviews the challenges in, and opportunities for, bringing a gender analysis together with social–ecological resilience analysis in the context of small-scale fisheries research in developing countries. We conclude that rather than searching for a single unifying framework for gender and resilience analysis, it will be more effective to pursue a plural solution in which closer engagement is fostered between analysis of gender and social-ecological resilience whilst preserving the strengths of each approach. This approach can make an important contribution to developing a better evidence base for small-scale fisheries management and policy.

## Introduction

The purpose of this paper is to analyse the challenges involved in bringing gender analysis together with social–ecological resilience analysis and, in doing so, to provide ways forward that will enable a meaningful account of gendered social relations in relation to social–ecological dynamics. The paper is based primarily upon a review of literature attending to both gender and small-scale fisheries in developing countries, but has also included other gender studies concerned with other ecological systems, natural resource management, adaptation and climate change. Our selection of references is informed by our critical judgement and our intention to illustrate significant directions in thinking. We also draw on our experience of working together to build capacity in gender research within WorldFish and the Aquatic Agricultural Systems Collaborative Research Program of the CGIAR from 2013 to 2015.

Whilst there are a plethora of terms and approaches connected with social–ecological resilience, our conceptual focus is on approaches to research that are based on the same set of fundamental concerns and logics about the capacity of *inter* *linked social and environmental systems* to adapt to environmental changes at various levels. For clarity, we refer to these system-orientated perspectives hereafter as ‘social–ecological resilience analysis’.^1^ Our approach to gender analysis is strongly embedded within critical social theory (Jackson and Pearson 1998; Kabeer 2000; Jackson 2006). We acknowledge that the challenges and opportunities identified are not necessarily exclusive to gender analysis but are often central to doing ‘good’ qualitative social science.

We begin by introducing the analysis of social–ecological resilience and examine the ways in which gender has been integrated into social–ecological resilience analysis to date. We move on to review literature on qualitative gender analysis in small-scale fisheries and discuss what it has had to say about social–ecological resilience. In the section, “Re-invigorating the encounter between gender analysis and social–ecological resilience analysis”, we suggest that the way forward lies in a closer engagement between plural analyses of gender and social–ecological resilience. We argue that for gender analysis to effectively enrich social–ecological resilience research it needs to be theoretically and methodologically rigorous. We conclude that fostering a richer conversation between gender research and social–ecological resilience research has the potential to generate a stronger evidence base for policies that facilitate adaptive strategies that are gender equitable and pro-poor.

## The analysis of social–ecological resilience and its engagement with gender

Social–ecological resilience is understood as the capacity for inter-related ecological and social systems to absorb or adapt to shocks or stressors without changing state (Walker et al. 2004). The concept was initially developed from resilience thinking that originated from the field of ecology. The recognition that ecosystems are complex, uncertain and dynamic (Holling 1973) changed the objective of ecosystem management from stability to building ecological resilience in order to deal with uncertainty and to adapt to changes. Human activities (e.g. fishing and aquaculture) were considered to be significant elements that affect ecological resilience, and therefore understanding social contexts became increasingly important for maintaining ecological resilience. In the late 1990s, the importance of understanding the interdependent relationships between ecological systems and social systems was accepted (Berkes and Folke 1998) and this laid the groundwork for opening up a new research agenda around social–ecological resilience (see Folke 2006 for a detailed account).

Social–ecological resilience thinking is a form of ‘systems thinking’ (Walker and Salt 2012: 11). It considers ecological systems and social systems as integrated analytical units, referred to as coupled social and ecological systems (SESs) (Berkes 1996, Berkes and Folke 1998), which are nested within powerful reciprocal feedbacks that operate across multiple scales (Gunderson and Holling 2002). It considers that human actions influence and are influenced by ecological systems, moving forward from looking narrowly at ecological production systems to greater recognition of the need to support local management institutions and local resource users to adapt to changes (Berkes et al. 2003). This paradigm shift helps find context-specific policy options for establishing flexible resource management approaches as alternatives to a universal management policy (Hughes et al. 2005). This idea is useful for fisheries and aquaculture policies in developing countries that need to consider the consequences of policy changes for the poor who depend heavily on natural resources.

In the 2000s, social–ecological resilience thinking evolved from a focus on adaptability to include some focus on transformability (Walker et al. 2004). Transformability refers to ‘the capacity to create a fundamentally new system when ecological, economic or social (including political) conditions make the existing system untenable’ (Walker et al. 2004: 3). This broader conceptualization has increased the dynamic nature of social–ecological resilience thinking in terms of the degree of change and kinds of outcome considered, including radical actions for future social–ecological well-being (Keck and Sakdapolrak 2013, p. 9). In this respect, transformation can also potentially be a progressive deliberate change that challenges existing power relations, shifting to pro-poor and more gender equitable systems. Whilst social–ecological resilience thinking has increasingly been used in this broader sense, social–ecological resilience researchers point out that efforts to bring together social and ecological analysis are very much in their infancy (Folke 2006, p. 264) and that a number of clear challenges have emerged (Stone-Jovicich 2015).

At the root of these challenges is that processes of social change or transformation are essentially different from those of ecological systems. In particular, this has manifested itself in difficulties for social–ecological resilience analysis in engaging with the inherent, complex, dynamic and sometimes conflictual power relationships that exist in society. This includes challenges in addressing the ways in which different groups of resource users are affected by shocks and adapt to change differently, and how individual agency and power relations mediate stasis or changes in the systems (e.g. Davidson 2010). Whilst there are increasingly sophisticated efforts to integrate social diversity and social power into social–ecological resilience research, “resilience thinking’s view of the ‘social’ is overridden by ecological understandings of system characteristics and dynamics” (Stone-Jovicich 2015, p. 25). Recognizing this, some critical social researchers have sought to develop the potential of social–ecological resilience analysis as a malleable cross-disciplinary approach (see Brown 2014), to positively address its capacity to analyse social dynamics (see Table 1).

**Table 1:** Variants of social–ecological resilience analysis addressing social dynamics

Approaches	Key papers	A unit of analysis	Objectives	Analysis of agency	The focus of analysis for understanding power	Understandings of Social change
Well-being	Brown and Westaway (2011), Coulthard et al. (2011) and Armitage et al. (2012)	Individual	Identifying subjective factors that shape people’s adaptive strategies	Yes	Intra-personal trade-offs	Mediated by individuals’ perceptions of well-being
Psychology & Mental health	Berkes and Ross (2013)	Community	Identifying subjective factors associated with community resilience	Yes	No	Mediated by personal, cognitive and spiritual factors and personal goals
Transition theory	e.g. Bush and Marschke (2014)	Community State Worldwide	Understanding the impact of technological change on the society and environment	Yes	Macro level	Mediated by socio-economic conditions, conflict of interest at multi-levels
Political ecology	Beymer-Farris et al. (2012), Turner (2014) and Nayak et al. (2014)	Social group	Understanding unequal distribution of costs and benefits in environmental change	Yes	Among different social groups	Mediated by social power
Network theory	Janssen et al. (2006)	Community	Identifying social–ecological networks and their effects on social–ecological resilience	No	No	Mediated by social networks

This table focuses only on attempts to *theorize* resilience analysis more broadly

Some social–ecological resilience research has begun to engage increasingly strongly with individual concerns around attitudes and psychologies, including people’s values, interests and perceptions of risk and well-being. This has helped social–ecological resilience research and adaptation research (de la Torre-Castro 2006; Brown and Westaway 2011; Coulthard et al. 2011; Coulthard 2012) unpack why people’s responses to change may not always appear rational in relation to the concerns of economics or ecology. In fisheries, for example, fishers rarely leave fisheries even when they recognize reduced fish catches and income; some cases, this is because fishing is central to their life satisfaction (Coulthard 2012). Further variables that have been identified as influencing people’s adaptive strategies include social ties, trust, identity, perceptions, aspirations and satisfaction (Armitage et al. 2012). These subjective and relational variables are very useful in understanding people’s decisions associated with potential trade-offs at intra-personal level, but do not explore negotiation processes and trade-offs at the interpersonal level (*between* individuals). De la Torre-Castro and Lindstrom (2010) investigate the complex interactions and conflicts that can arise when ‘slow-moving’ normative and cultural-cognition values are at odds with ‘fast-moving’ regulatory changes in Chwaka Bay, Zanzibar. Increasingly, attention to institutions has been orientated towards quantitatively modelling how far existing social relations constrain or enhance the potential for adaptive management (such as Bodin et al. 2006). And yet, such studies miss the way in which gendered power relations constrain the potential for social capital to deliver equitable change (Cleaver 2005) as well as the way in which gendered coping mechanisms are embedded in existing (unequal) systems (Overå 1993).

Studies drawn from political economy and political ecology do focus on the role of power and show that a small number of elite actors—generally powerful men—tend to take advantage of processes of environmental or policy change to further their benefits from natural resources and strengthen their influence over the social and ecological system within which they are embedded (Nadasdy 2005; Neiland et al. 2005; Russell and Dobson 2011). Some studies posit that whilst those who have economic or political power exploit natural resources in their own interests, those who use natural resources in sustainable ways are often excluded from the new system (Adduci 2009; Sneddon and Fox 2012). Conversely, Onyango and Jentoft (2010) show how poverty can pose a different set of challenges for the governability of small-scale fisheries: their study of Lake Victoria shows that strong social values that uphold poor fishers’ rights to feed their families prevent villagers from regulating one another’s fishing. These studies directly address power relations played out in the processes of change and highlight unequal exchange among the people in the same system. Where the primary analysis focus is social–ecological resilience, these studies have not attended to gender relations.

While eclectic in origin, these have all included attention to social relations, either implicitly or explicitly. However, none of the above approaches to social–ecological resilience analysis include specific attention to gender. Indeed, the absence of analytical attention to gender in social–ecological resilience, and the many reasons for it, has been extensively noted (see Cote and Nightingale 2012; Fröcklin et al. 2013, 2014; Keck and Sakdapolrak 2013; Stone-Jovicich 2015). Here we draw attention to the way in which gender analysis and social–ecological resilience analysis are rooted in fundamentally different epistemologies and methodologies. The central analytical impulse of gender analysis is one of critique, in which inequality is a central trope and where the case built is one that requires redress (Jackson and Pearson 1998; Jackson 2006; Cornwall et al. 2007). In contrast, the central analytical impulse of social–ecological resilience analysis is one of complex causal explanation, in which the modelling of coupled systems in terms of critical factors, dynamics and thresholds is a central trope and where the case built is one that predicts adaptation or transformation and calls for action to trigger, facilitate or avoid this (Table 2). These differences are problematic in trying to develop a unitary framework for gender analysis and social–ecological resilience analysis, raising difficulties about how to reconcile understandings of change and ways of finding out about these changes.

**Table 2:** Differences between gender analysis and social–ecological resilience analysis

	Gender analysis	Social–ecological resilience analysis
The relevant disciplines	Feminism, Critical social theory Critical intellectual practice	Ecology Interdisciplinary practice
The analytical concern	Social inequality in gender relations that influences the processes of social change.	The coping, adaptive and/or transformative capacities of actors, communities and larger systems.
The aims of analysis	Critical explanation: understanding the processes of change and how gendered agency and power relations play out in the processes.	Complex causal explanation: identifying non-technological and non-environmental factors that facilitate or impede system change.
Core methodologies	Providing in-depth descriptive information, often informed by ethnography and political science. Critically reflective, context-specific and interpretive.	Using models as a tool for understanding what works in helping social–ecological systems manage stresses and shocks effectively.

Despite these challenges, the importance of a gender lens in small-scale fisheries has been well recognized (Bennett 2005; Choo et al. 2008; Williams 2008) and research into small-scale fisheries has sought to include gender in its analysis of social–ecological resilience. However, Carr and Thompson (2014) point out that when gender is integrated into social–ecological resilience frameworks, it tends to be considered as a variable. This results in a focus on understanding gender differences in access, roles, management and decision-making related to natural resources, in order to enumerate the ‘gaps’ between men and women. This is a step forward for social–ecological resilience analysts in identifying causal relationships between gender inequality and the extent of social–ecological resilience at community or household levels. It also provides some value for the basic targeting of interventions (Locke and Okali 1999, p. 283) and facilitates a straight-forward design for impact assessments (Carr and Thompson 2014, p. 191). Nevertheless, the literature reviewed suggested that ‘mainstreaming gender’ in social–ecological resilience analysis has been seen largely in terms of identifying what ‘additional’ data need to be collected to enhance existing analyses .^2^

Integrating gender as an additional variable lacks the social theoretical content that is needed to open up space for critical analysis (Rocheleau 2008). Specifically, it does not address the question of *how* people occupying different gender positions negotiate around the natural and other kinds of resources that they share, or of *how* this plays out in the different ways that they are affected by, and able to respond to, shocks (Kaijser and Kronsell 2014). Accordingly, the next section now turns to review literature on gender analysis with references to small-scale fisheries and discuss how far gender analysis has informed social–ecological resilience to date.

## Gender analysis in small-scale fisheries and insights for social–ecological resilience

A review of the existing literature signals three areas of learning relevant to understanding gendered social relations embedded in fishery-based livelihoods: masculinities and gender relations in fishing communities; gender and social vulnerability in fishing communities; and feminist political ecology and closely related contributions in human geography. For each of these, we ask what we might learn from these gendered studies for social–ecological resilience analysis and what limitations these studies have in terms of opening up understanding about social–ecological resilience. The conclusion parallels the above regarding gender in resilience analysis: while the studies considerably deepen our knowledge about gender in relation to the natural environment, as a whole they stop short of engaging directly with ecology, remaining for the most part centred within the social domain.

### Research on masculinities and gender relations in fishing communities

Although there is a rich literature on the role of masculinities^3^ and gender relations in fishing communities, it has been largely dissociated from thinking about or analysis of social–ecological resilience. Masculine identity has featured prominently in the anthropological exploration of the culture of fishing communities. Small-scale fishing—as a high-risk and individualized occupation, with highly variable cash returns, and which often encompasses a high degree of mobility (Fabinyi 2007; Geheb et al. 2008; Mojola 2011)—has often been associated with the dominance of masculine identities that value men’s risk-taking and a sharp distinction of gender roles, fuelling social problems around alcohol consumption, violence and risk-taking sexual behaviour (Cardoso 2002; Allison and Seeley 2004; Ford and Chamratrithirong 2008; Tumwesigye et al. 2012). For example, Cole et al. (2015) explore how fishermen’s masculinity in the Barotse flood plain in Western Zambia shapes gendered spending practices and imposes additional obligations and responsibilities on women. These studies offer some appreciation of how ecological shocks and stressors influence the ongoing construction of masculinities, but tend not to engage directly with ecology.

Research on gender relations in small-scale fisheries has increasingly illuminated women’s involvement in fishing (Kleiber et al. 2015), in their ‘invisible’ support for men’s fishing (Bennett 2005), and at different stages in the fish value chain (Fröcklin et al. 2013), and in mariculture (Fröcklin et al. 2012). Studies of informal fish trading are a rich source of information that illuminate gendered agency and power dynamics and their variations. These studies show the dangers of universalized generalizations and reveal that gender relations are highly context dependant. Small-scale fisheries often involve reciprocal relationships in the processes of production, trading and marketing between boat owners and their male fishers, male retailers and female processors, and fishermen and female traders (Overå 1993). Although unequal, these relationships can be the basis on which poor men and women negotiate and mobilize resources in times of need to cope with difficulties and to maintain their livelihoods (Walker 2001; Gordon 2006; Merten and Haller 2007; Lwenya and Yongo 2014; Kawarazuka 2015). Fröcklin et al. (2014) is unusual in linking a close analysis of gender roles and interests with a detailed ecological assessment of tropical invertebrates in Chwaka Bay, Zanzibar: the researchers use this analysis to draw out the gendered dynamics around (un)sustainable development of this fishery.

The implication drawn from these studies is that capacities to adapt, either individually or collectively through co-management institutions, and adherence to fishing regulation, are not only significantly affected by fishermen’s income and well-being, but also by gendered social relations, as well as vice versa (Nunan et al. 2014). Whilst questions of ecology are rarely addressed, these studies illuminate more clearly the gendered dimensions of resource-based livelihoods and reveal how they may, or may not, be congruent with ecological resilience. As such, these studies offer rich qualitative data that contribute to the aims of social–ecological resilience analysis by generating a better understanding of gendered negotiations around adaptation for different individuals.

### Research on gender and social vulnerability in fishing communities

Studies concerned with gender and social vulnerability offer rich insights into fishing communities. These emerged particularly since the 2000s after the recognition that HIV infection rates in fishing communities in some low- and middle-class countries in sub-Saharan Africa, Asia and Latin America were much higher than national average prevalence rates (Kissling et al. 2005). High infection rates were understood as resulting from gendered social norms and practices in informal fish trading between (migrant) fishermen and female traders (e.g. Allison and Seeley 2004; Béné and Merten 2008). One consequence for many affected fishing villages was inevitably that their economic and social capacities to respond to change, including environmental change, was low. The pertinence of these studies extends beyond analysing situations of high HIV-risk to other highly vulnerable fishing communities and has opened up a much more sophisticated exploration of the importance of gender relations and social vulnerability in small-scale fisheries characterised by widespread poverty (see for example, Nunan 2010)

Within this literature, Merten and Haller’s study (2007) in the Zambian Kafue flats has a particular salience. Ila women, formerly agro-pastoralists, started trading fish because their incomes from maize fell and by negotiating directly with the Lozi fishermen (on the shore or at the fishermen’s houses, instead of at the fish markets) they were able to sustain their activities even during the season when fishing was officially prohibited. Furthermore, some poor women with limited capital accessed fish from the fishermen in exchange for sex, a practice called ‘fish for sex’. The Ila women legitimized ‘fish for sex’ by constructing it as *lubambo*, an old customary regulation of extramarital sexual relations through which women used to fulfil their material needs in times of need. The authors closely explore how women constructed, exercised and renegotiated their decisions to engage in ‘fish for sex’. Although the study does not directly address the influence of these on ecological systems, it elucidates the way in which changes in wider gender relations and fisheries livelihoods are mutually interlinked in context-specific ways that implicate not only “reproductive roles, such as childcare and household responsibilities” (Fröcklin et al. 2013) but also sexual and conjugal strategies. In this way, gender research on HIV and AIDS has contributed to explaining gendered vulnerability in some marginalized fishing societies. It provides a complex picture in which the gendered exercise of agency interplays with wider or external threats such as environmental and economic changes with deeply ambiguous implications for both ecological sustainability and human well-being.

### Feminist political ecology and related approaches in human geography

In contrast to other forms of gender analysis, feminist political ecology (FPE) has directly attempted to engage with the indivisibility of social and ecological systems and is credited with making a valuable contribution to the broader political economy through its sophisticated engagement with power and agency. Whilst we found no self-identified FPE of small-scale fisheries, there has been a rich strand of analysis focusing on other common-pool natural resources, and particularly on forests. For example, in her case study of forest conservation in Nepal, Nightingale (2006) showed how the forest resource is central to producing and reproducing social inequality and that women’s gendered agency around forest exploitation serves to sustain existing social inequality as well as resist new resource management practices. A recent resurgence in FPE (Elmhirst 2011a, p. 130) has argued for a shift in analytical focus from women, or other specific social groups, to interdependent and dynamic power relations within family and community (Nightingale 2011; Truelove 2011; Elmhirst 2011b). For example, Resurreccion and Elmhirst (2008) explore “how gender subjectivities, ideologies and identities are produced, employed and contested within natural resource governance” (3) whilst Elmhirst (2011b) explains how locally recognized masculinities and conjugal relations influence forest management in Indonesia.

Aside from studies labelled as FPE, there are many studies that sit broadly within human geography that relate closely to the concerns and approaches of FPE (Elmhirst 2011a). Although not self-identified as FPE, Resurreccion’s study in the Tonle Sap Great Lake in Cambodia (2008) is closely informed by gender theory and explores power relations over a shift from male-dominated traditional fishery management to a newly formulated management institution in which women are involved. She found that women legitimize their position in the management institution and benefit from the management programmes through influential male relatives. In this way, the new co-management system is traditionalized and reproduces male power and authority. Her case study demonstrates the complex ways in which gendered power relations shape processes of environmental and institutional change and asks direct questions associated with environmental concerns.

To sum up, the studies reviewed above may still frustrate or be distanced from social–ecological resilience researchers in that they are not orientated towards identifying social–ecological solutions or developing more effective models. In this sense, both their complexity and ambiguity can be unsettling. Moreover, despite their close engagement with natural resource use and governance, none of these studies effectively counters the question that social–ecological resilience scholars have asked, namely “‘where is the ecology’ in social analysis?” (Stone-Jovicich 2015, p. 25). Indeed, Peterson carefully evidences how FPE, and political ecology more broadly has largely been feminist political *economy* and has failed to say anything about ecology, or about the feedbacks to and interactions of social ‘systems’ with ecological ones (2000, p. 234). Thus, whilst gender analysis in small-scale fisheries (and more broadly in relation to other natural resources) has made progress with understanding gendered social dynamics and individuals’ gendered adaptive strategies in relation to natural resources, it has failed to engage directly with the resilience of environmental and ecological systems.

The above underscores that thus far, it has proven challenging to develop a meaningful engagement of the social relations and gender in relation to social–ecological resilience (Cote and Nightingale 2012; Harrison and Watson 2012; Keck and Sakdapolrak 2013). Whilst many valuable insights have been generated, there is as yet no unifying or mutually acceptable framework or approach to act as a ‘bridge’ to connect these two important fields of research. Moreover, the epistemological and methodological differences suggest that such a unifying framework may be unlikely. If this is the case, going forward, what are the possibilities for a closer engagement?

## Re-invigorating the encounter between gender analysis and social–ecological resilience analysis

We have argued that epistemological and methodological incompatibilities between gender analysis and social–ecological resilience analysis mean that gender concepts are often stripped of theoretical content when they are integrated into social–ecological resilience analyses.^4^ Whilst the ‘integration’ of gender as a variable into ongoing social–ecological systems research on resilience in small-scale fisheries, is both desirable and necessary, it cannot, on its own, achieve what is needed. Indeed, as Bennett (2005, p. 451) notes, it is “an understanding of *the complexity*” (emphasis ours) of gender relations and their “nuances” that are needed to better inform policy-making for fisheries management. Conversely, the above showed that when the strengths of gender were central, ecological issues tended to fall aside. The challenge is thus to enable the respective strengths of both gender analysis and resilience analysis to be sustained, whilst working to extend and deepen their mutual engagement with one another.

So, rather than seeking a single unifying framework for gender and social–ecological resilience analysis that works for small-scale fisheries, we suggest instead fostering the basis for a closer interdisciplinary engagement between social–ecological resilience analysis and gender analysis in small-scale fisheries research. A plural research strategy to develop this engagement could combine: setting the research agenda in a purposefully interdisciplinary way; continuing the ongoing effort to increase and improve the collection of sex disaggregated data in ongoing small-scale fisheries systems research; and, adding further emphasis on developing high-quality gender analysis on questions related to social–ecological dynamics in small-scale fisheries.

There is substantial and ongoing progress that is being made with disaggregation. This is particularly the case where the collection of binary data on men and women has been further differentiation by intersecting variables such as age, class, caste and household headship (e.g. Huynh and Resurreccion 2014), thus addressing the long-standing critique that men and women are not homogenous groups (e.g. Kandiyoti 1998). Accordingly, we devote the rest of our attention in this paper to the other elements of this strategy, namely the proposal for interdisciplinary agenda setting, and that of fostering high-quality gender analysis in small-scale fisheries. Below we begin by proposing that interdisciplinary engagement begin with the framing of research questions of mutual interest. We then proceed to highlight three theoretical and two methodological principles of gender analysis that have considerable potential to add value to interdisciplinary research but which are often ‘lost’ in attempts to integrate gender into social–ecological resilience analysis or social–ecological frameworks (Diamond et al. 2003; Cote and Nightingale 2012; Keck and Sakdapolrak 2013).

### Securing an interdisciplinary research agenda for gender and social–ecological resilience analysis in small-scale fisheries

To address the challenges of gender-based research that has struggled to engage with ecological issues, we propose purposeful engagement between the disciplines that begins at the problem analysis and question-setting phase of research. Formulating overarching questions that are firmly rooted both in critical gender theory and the ongoing concerns around social–ecological resilience lays the foundation for the type of research practice that can effectively engage with complex fisheries issues. This agenda-setting process could begin with joint agreement of sets of questions that are of *mutual* interest to both gender researchers, social–ecological resilience researchers and other stakeholders (see also Locke and Okali 1999). This joint framing of questions can provide vital direction for analysis and interpretation: securing the relevance of gender research to those primarily focused on understanding social–ecological change, and conversely, ensuring that gender researchers explicitly engage with important ecological dynamics.^5^ The identification needs to be grounded in a good appreciation of existing knowledge about gender and environment in specific contexts, thus providing a valuable briefing for a multi-disciplinary team, adding depth to the delineation of context-specific questions, and providing essential context for interpreting data. Importantly agreeing research questions is not the end point of such an approach—the discussion of findings, debates over their interpretation in relation to context-specific concerns around social–ecological resilience, and their meaning in relation to the wider fields of knowledge about gender and natural resources all need to be seen as core activities for successful interdisciplinary engagement.

### Retaining the theoretical principles of gender analysis

Firstly, quality gender analysis that considers individuals’ capacities to adapt to change can move beyond the analysis of gender ‘gaps’ to consider how interdependent gender relations work. Interdependency is intrinsic to gendered power relations and therefore it can be used by the marginalized for negotiating their position in their favour (Connell 2009). Women often leverage gendered relationships: appealing to the sympathies and loyalties of immediate and wider natal and marital kin, friends, community groups and leaders or other patrons. Exploring the interdependency of relations between unequal individuals, households and groups makes visible the ways in which less powerful people exert gendered agency in their negotiations. For example, some poor fishermen sustain fishing activities through negotiations with more powerful fishermen for instance over species to be targeted or over fishing areas (Overå 1993), and likewise, female traders may sustain access to fish through renegotiating their relationships with particular fishermen (Merten and Haller 2007, Kawarazuka 2015). Critical gender analysis focuses on the trade-offs and tensions in interdependent relationships, that involve both cooperation (and joint interests) and conflict (and individual interests), among men and women in different social positions (Kabeer 2000). This more sophisticated analysis of the ways in which human agency is profoundly imbued with power relations (Davidson 2013, pp. 22–23) is valuable for those trying to influence or understand behaviour in small-scale fishing communities. It is also useful for understanding how institutional changes for managing social–ecological systems may impinge on unequal exchanges, potentially making some groups of people more vulnerable (Hornborg 2009).

Secondly, critical gender analysis that contextualises changing fishery resource behaviours within a wider web of dynamic gendered social relations can offer a fuller exploration of change and its implications. Changes in gendered power relations in a specific fishing community or industry may impinge on changes in fisheries management and vice versa, changes in fishing stocks or their management can impinge on changing gender relations. These wider gender power relations and the specific gender power relations around fishing are closely intertwined: both are generated and sustained through everyday practices, with changing practices resulting in changing power relations (Connell 2009).

In the context of small-scale fisheries, everyday routine practices such as fishermen going to fish, interacting with female traders and giving cash to their wives, contribute to sustaining the existing gendered power relations. Consequently, men may resist changing practices to sustain their power while some adaptation strategies result in changing the existing power relationships, influencing the interdependent relations through which poor men and women ensure security and maintain their well-being. Therefore, fishermen’s decisions with respect to changes in their livelihoods, and thus their means and processes of adaptation, are not made simply according to whether they have alternative economic livelihoods or whether they place a high value on fishing as a man’s job, but *also* with respect to how this might affect their prospects for marriage, their position as husbands or fathers, their support of their younger brothers, their standing in the fishing cooperative or the security of their sales to specific female traders. This broader calculus inevitably strays way beyond the natural resource (Bennett 2005) or ecological system of interest to resilience researchers, but by doing so it offers a “clearer understanding of the linkages among gender equality, natural resource management and sustainable development” (Brewster 2004, p. i).

Thirdly, gender analysis that moves beyond seeing norms as ‘rules’ determining or constraining behaviour, can examine how context-specific meanings and ideas are deployed in ongoing negotiations over fisheries, often in subtle or ambiguous ways. In any context, there are wide variations in actual gender practices which in many situations are ‘hidden’ under a veneer of consensus over hierarchical gender ideologies (Kabeer 2000; Connell 2009). A rigorous account of gender needs to combine actual observation of behaviours (empirical analysis) with what people say about gender (narrative analysis) to gain critical purchase on what gender norms really mean for gender relations. For instance, Kawarazuka (2015) shows for coastal Kilifi in Kenya that young women often prioritize cooking for a reliable husband and his friends over fish processing to earn income *because* doing so demonstrates that they are ‘good wives’ enabling them to gain bargaining power within a marriage that is central to their long-term security. This ‘bargaining with patriarchy’ (Kandiyoti 1998) is highly strategic and illustrates the importance of understanding how and why different men and women are invested in existing practices and beliefs as well as the reasons why they may seek to change, retain or renegotiate these in the face of ecological shocks, stressors or changing management regimes.

To conclude, applying critical gender analysis will not directly achieve the aims of social–ecological resilience analysis, but it will powerfully deepen the appreciation of what different possible social–ecological change might mean and for whom. It can also add depth to understand the changing negotiations around changing common-pool resource use and management, and interpret what this means for gendered power relations, and the resulting social–ecological resilience, vulnerability and ‘room for manoeuvre’ of different men and women arising from these dynamics. This can contribute to shifting the emphasis of social–ecological resilience research (Anderies et al. 2006) towards a field of debate that “opens up issues around values,… equity and justice” in order to “formulate questions about which resilience outcomes are desirable, and whether and how they are privileged over others” (Cote and Nightingale 2012, p. 480). This will provide a strong common ground for starting new conversations about how interventions designed to enhance social–ecological resilience may be linked to gendered social relationships and changes in gendered power relations. Delivering a theoretically rigorous account of gender is methodologically challenging, so we now turn to three suggestions that we believe are key for delivering an empirically rigorous account of gender analysis for small-scale fisheries.

### Improving the methodological rigour of gender and SES analysis for small-scale fisheries

Firstly, rigour in all qualitative methodologies is intrinsically reliant on the field researcher’s engagement with the underlying aims of the enquiry and critical thinking about researchers’ relationships with respondents are central (Rose 1997; Jackson 2006). The former is central to doing ‘good’ qualitative research and requires deep engagement between senior researchers and a small skilled team of researchers involved from design through to interpretation.^6^ This latter enables a proper reflection on how a researchers’ positionality affects their relationships with respondents and mediates their answers to questions (e.g. Callaway 1992). Findings from qualitative research are shaped by the positionality accorded to researchers by local people and the specific narratives that respondents offer are tailored towards those they feel will make sense to the researcher (Rose 1997). Research teams need to record their ongoing reflections on these dynamics and take them into account in the analysis of the data.

Secondly, avoiding an over-reliance on participatory methods and including methods that are better at probing gendered power relations is central to effective qualitative research. Participatory approaches have been the dominant method for qualitative research in relation to social–ecological systems, in part because they fit well with intervention strategies seeking to foster co-management and adaptation (for example, Armitage et al. 2011). However, participatory methods neglect the way in which gendered power shapes the production of knowledge in participatory processes. Johnson et al. (2004) note that participatory research in natural resource studies tends to lag behind ‘best practice’ (2004, p. 189) and “may be particularly unrepresentative of the priorities and concerns of marginalized groups” (2004, p. 198). Where NR researchers have acknowledged these problems, for example, Pohl et al. (2010), there is a tendency to try to resolve them technically by focusing on how to organize workshops and build relationships with participants. Even where ‘better facilitation’ of participation penetrates the reticence or silence of marginalized people in collective fora, what they say in these contexts is necessarily mediated by judgements about what is politic or desirable to be expressed in public (Mosse 1994). The meaning and significance of these narratives need careful interpretation in relation to other kinds of data generated using alternative methods (Jackson 2006). Diamond et al. (2003) note that for effective gender research, participatory methods are simply not enough. Methods that are better at revealing what is ‘hidden’ are valuable antidotes to participatory and focus group discussion methods. Ethnographic observation, life history research and open-ended in-depth interviews all allow the space for researchers to build up a much more nuanced account of the workings of gender relations around specific events or processes and in relation to complex social–ecological phenomenon.

To sum up, joint agenda setting and gender analysis that maintains its critical edge and methodological rigor can make significant contributions to critical analysis around shared challenges of social–ecological resilience in targeted communities. These kinds of contributions can powerfully animate the strengthened collection of gender disaggregated data in social–ecological resilience analysis, and as a result will add depth to understandings of how gender relations in specific contexts relate to cases of social–ecological crisis, adaptation or transformation. In doing so, this strengthened engagement of critical gender analysis and social ecological resilience can add value to understanding the interaction of society with ecological systems, and can contribute to ongoing debate about resilience of what and for whom.

## Conclusion

Our review of the challenges and opportunities of bringing gender analysis and social–ecological resilience analysis together in small-scale fisheries concluded that there are fundamental constraints to developing a satisfactory unifying framework for gender and social–ecological resilience analysis. Indeed, “The concerns and questions raised by both resilience scholars and social scientists are, at base, reflections of very old and enduring tensions and debates within and across the natural and social sciences” (Stone-Jovicich 2015: 25). Despite significant progress and important insights on both sides, two key constraints emerge in existing research that attempts to bridge this divide. Firstly, attempts to integrate gender into social–ecological resilience analysis are weakly engaged with gender theory or methodology; and secondly, that gender analysis of fisheries has yet to move beyond the social domain to really engage directly with questions of ecology, which can better inform resource management.

Consequently, we have argued that the goal of bringing gender analysis and social–ecological resilience analysis together need not be a search for a unifying framework but instead could be seen as a quest to deepen interdisciplinary engagement over social–ecological resilience. In this sense, we very much follow Jovicich’s invocation to build ‘disciplinary depth’, although we depart from her goal of building a ‘transdisciplinary synthesis’ (Stone-Jovicich 2015: 24), in favour of closer interdisciplinary engagement. As such, we have argued that it is important that gender research addressing social–ecological dynamics needs to explicitly and deliberately deploy critical social theory. This refocusing means that it is the generation of deeper insights about gender and social–ecological dynamics, and not whether these can be subsumed by ‘a’ social–ecological resilience analysis or by ‘a’ gender analysis, which matters. The desired outcome becomes a much strengthened critical debate over different processes of social–ecological change and their interaction with changing gendered power relations. In this way, the undertaking is about carving out a more plural space for mutually constructive debate.

Such an engagement has the potential to add value to gender analysis and social–ecological resilience analysis, respectively. Gender analysis would be enriched by asking questions about how unequal gender relations are invested in, are challenged by, or are contributing to changing existing social–ecological systems. Social–ecological resilience analysis would be enriched by asking questions about how experiences, priorities and adaptation capacity in the face of ecological shocks and stressors are shaped by, and in turn shape, gender inequalities. Where gender analysis would gain from analytical tools that focus on complexity, surprise and adaptation, social–ecological resilience analysis would gain from an analytic emphasis on tensions, trade-offs, conflicts and ambiguities.

Most importantly, though, bringing critical gender analysis and social–ecological resilience analysis into conversation has the potential to generate powerful understandings of integrated social and ecological systems. These are not only vital for making progress in enhancing the rigour of social–ecological research but are also valuable in generating a better evidence base for policy-makers in small-scale fisheries and other ecological systems who are faced with increasingly urgent decisions about adapting to climate change.

## Footnotes

1 There are other forms of resilience analysis that are not systems based and these are not the focus of our enquiry. Nor are we focusing here on ‘social resilience’ which is usually employed to describe social dimensions at the level of the individual, household or community, without consideration of impacts on ecological or natural resource systems (for further commentary on social resilience see, for example, Marshall et al. 2007).

2 This story is by no means unique to fisheries research on resilience, and is an all-too-common feature of the history of gender mainstreaming in research (Cornwall et al. 2007).

3 Masculinities are about what it means to be a man in a particular gender order: as such, masculinity is not men themselves, but rather is about the practices and identities of being a man in a particular time and place (see Connell 2009).

4 This process parallels that loss of critical edge that occurs when social concepts are ‘naturalized’ in social–ecological analysis (Bush and Marshke 2014, p. 49).

5 This has the potential, for example, to shift a research question like ‘how resilient is x small-scale fishery to changing climatic conditions?’ in the direction of, say, ‘how do different men and women strategise around changing climatic conditions in x and what does this mean for them, their families and the fishery respectively?’.

6 Key elements of ‘good’ practice for such teamwork include: fully enrolling fieldworkers in the critical aims and design of the enquiry so that they can attend to and probe the relevant issues in the field and engage in critical discussion of the meaning of resulting data; additional notes on the context, participants and ‘feel’ of each interview, conversation or observation that go beyond verbal interactions and which are taken into account during interpretation; recorded reflections (often in the form of research diary) on the research process as it unfolds and direct involvement of fieldworkers in verifying the analysis and interpretation of data in research outputs.
